# Otolith chemistry suggests population heterogeneity within a genetically homogeneous Indian scad population along Indian coast

**DOI:** 10.1038/s41598-025-85719-3

**Published:** 2025-01-08

**Authors:** Anjaly Jose, Sandhya Sukumaran, Neenu Raj, K. Nisha, Eldho Varghese, S. J. Laly, Satyen Kumar Panda, Subal Kumar Roul, P. Abdul Azeez, Shoba Joe Kizhakudan, A. Gopalakrishnan

**Affiliations:** 1 Marine Biotechnology Fish Nutrition and Health Division, ICAR-Central Marine Fisheries Research Institute, Ernakulam North P O, Kochi, Kerala 682018 India; 2 Mangalore University, Mangalagangotri, Mangalore, Karnataka 574199 India; 3 ICAR-Central Institute of Fisheries Technology, Kochi, Kerala 682029 India

**Keywords:** Element/Ca ratio, Fish stock, ICP-OES, Management, Otolith chemistry, Sagittae, Biotechnology, Evolution, Genetics, Molecular biology, Zoology, Ecology, Environmental sciences

## Abstract

The Indian scad, *Decapterus russelli* is one of the most exploited pelagic resources of India. Population genetic analyses using mitochondrial and nuclear markers indicated a lack of genetic structuring among populations from Indian waters. As this species is highly migratory, it is also important to establish the environmental influence on its population structure. In the present study, the whole sagittal otolith composition was analysed using Inductively Coupled Plasma Optical Emission Spectroscopy (ICP-OES) to assess spatial distribution of *D. russelli* collected from 4 sites along the Indian coast. Elemental ratios (Ba/Ca, Fe/Ca, K/Ca, Mg/Ca, Na/Ca, Sr/Ca and Zn/Ca) were analysed using univariate and multivariate statistics to determine whether this chemical signature can provide insight into the adaptive patterns. All element/Ca ratios are found to be significantly different between the four sampling locations (ANOVA, *p* < 0.05; MANOVA, *p* < 0.05). Five of the element/Ca ratios were found to be significantly different when the data was analysed coastwise (ANOVA, *p* < 0.05; MANOVA, *p* < 0.05). The LDA plot also showed the spatial heterogeneity of Element/Ca ratios between the four sampling sites, but some overlaps were also observed, reflecting the migratory ability of the species. This basic information on spatial ecology is required for formulating effective management and conservation strategies for the species.

**Supplementary Information:**

The online version contains supplementary material available at 10.1038/s41598-025-85719-3.

## Introduction

The Indian scad, *Decapterus russelli*, is a widely distributed pelagic species inhabiting the Indo-Pacific region, spanning the Red Sea and East Africa to Japan and Australia^1,2^.

It is a vital fishery resource in India and Southeast Asia and is one of the most important pelagic resources after the Indian oil sardine^3^. This species plays a significant role in food security as an affordable source of animal protein and is commonly used as live bait in fisheries^4^.

Ecologically, *D. russelli* serves as an essential link in the marine food web, functioning as a primary carnivore with a pronounced shift in dietary preference as it grows. Smaller individuals predominantly consume planktonic crustaceans like *Acetes spp.* and copepods^5^, while larger individuals feed on fish (e.g., *Lactarius spp.*, *Myctophid spp.*) and molluscs^6–8^. This dietary versatility helps maintain ecological balance by regulating plankton populations, preventing the over-exploitation of primary producers, and supporting the energy transfer across trophic levels. Additionally, the species’ dynamic feeding habits increase prey diversity and mitigate interspecific competition, contributing to ecosystem stability^5,6^.

Despite its ecological and economic importance, research on *D. russelli* has been limited and primarily focused on aspects such as biology^9^ population dynamics^7,10^, length-weight relationship^11^, food and feeding habits^6^, reproductive biology^5,12,13^, and population status^14,15^. The present authors have initiated genetic studies on this species from the Indian Ocean by characterizing the complete mitochondrial genome^4^. Population genetic analyses using mitochondrial and nuclear markers revealed panmixia in the Indian Ocean^16^. We tried to generated one more layer of information regarding population structure employing otolith-based elemental composition analysis. Combining genetic and otolith analyses is particularly significant for migratory species like *D. russelli*, as it provides a robust framework to detect subtle population structures and verify connectivity across spatial scales. This approach provides a comprehensive understanding of population structure, offering insights into the role of ecological factors shaping its spatial distribution.

By combining multiple markers, our study not only advances knowledge of *D. russelli*’s population structure but also highlights the importance of multi-faceted approaches for managing marine species. These findings are essential for developing conservation strategies and ensuring the sustainable management of this critical fishery resource.

Molecular genetic markers have long been a key tool to determine the geographical structure of fish population for several decades^16–19^. In some cases, the genetic techniques possess some limitations in resolving the task. Low levels of larval or adult mixing among populations may sometimes go undetected by genetic markers^20^. In addition, it cannot directly measure the individual exchange rate between sites or their origin^21^. Furthermore, though genetic differences between populations indicate little dispersal, sometimes the lack of difference is likely to be uninformative^22^. These difficulties associated with genetic markers have prompted more research for alternative and more definitive tool for assessing population structure of marine species and lead to the analysis of chemical composition of calcified structures^23,24^. In many cases, the chemical analysis of otolith has resolved several uncertainties on natal habitats and stock structure in both marine and freshwater environments, that the genetic markers were unable to address^20,25,26^. Recently, several studies have been conducted that coupled otolith chemistry and genetic analyses and have shown sometimes contradictory^27^ and complementary^28^ results revealing natal origin of individuals, habitat use and regional level population structure^29,30^. This was made possible by the fact that, in contrast to population divergence detected by genetic markers, which usually requires a longer time scale, otolith chemistry can be used to identify specific traits or events that have occurred within the time scale of an individual’s lifetime. Therefore, it is necessary to combine multiple methodological approaches to fully comprehend the spatial ecology and management requirements of a fish species and to influence local management practices. Employing multiple approaches to analyse the movement pattern and population structure is sometimes essential in case of migratory animals^31^.

Otoliths have been a key focus for fishery biologists and ecologists for decades, primarily for age and growth analysis through micro and macro structure examination^32–34^. Over time, otolith elemental analysis has become crucial in assessing fish stocks, delineating stock structure, and understanding fish population connectivity, providing valuable insights for fishery management^20,25,26^. Otoliths act as natural markers due to two key features: they are metabolically inert, and they reflect the physical and chemical environment the fish inhabit^35^. The incorporation of elements into the otolith is influenced by multiple factors^36,37^ and most are primarily affected by environmental conditions and serve as environmentally influenced stock markers^38^.

Various environmental process that influences otolith composition have been extensively studied^39–41^. The relationship between element and environmental process is often studied using element to calcium ratios (element/Ca) since the elements can replace Ca in CaCO_3_ matrix^42^. The advantage of using the chemical signatures of otoliths is that they can reveal the spatiotemporal environmental gradient that the fish experience throughout their lives^43^. The elemental chemistry of the otolith has been successfully used to elucidate the population structure of several fish species, even in systems with high gene flow where environmental heterogeneity exists^44–46^ and thus provide useful information about population dynamics^43,47^, movement patterns and habitat connectivity^48^.

There is no a priori reason for considering the elemental fingerprint as an indicator for population identity, even when there are no genetic differences. Besides, just as the environment at a particular location will not stay steady over time, there is no reason to believe that the otolith fingerprint of fish will be consistent over a long period of time. Nonetheless, because the otolith grows continuously, the elemental fingerprint of the otolith integrated over its lifetime can be used to distinguish fish that have been exposed to different environmental conditions^26^. Using otolith elemental fingerprint as a natural marker would exploit the fact that freshly added otolith material contributes to only a small proportion of the total otolith mass. Therefore, it is reasonable to assume that the changes in the environment and otolith growth during the (brief) period of migration or mixing will have very little impact on the fingerprint^25^. In principle, fish should be able to be identified as belonging to their source group until their elemental composition has been changed by subsequent otolith growth, provided that all possible source group are defined before the time of mixing or migration^21,49^. Analysing the otolith core is essentially used as a direct measure of stock or nursery origin^49^. Whole otolith fingerprints, on the other hand, may be applied more robustly to track stock migrations or stock mixing in natural populations^50^. Otolith elemental composition may therefore function as a stock identity marker as long as fish population or stocks inhabit different environmental conditions. In these scenarios, the fingerprints serve as biological tracers or natural tags for pre-defined fish groups over brief time periods^51,52^. The insights gained from such studies on the geographical structure of the population of any species is fundamental in understanding the nature and dynamics of their population, hence developing strategies for their management.

## Results

### Single elemental analysis

**Fig. 1:**
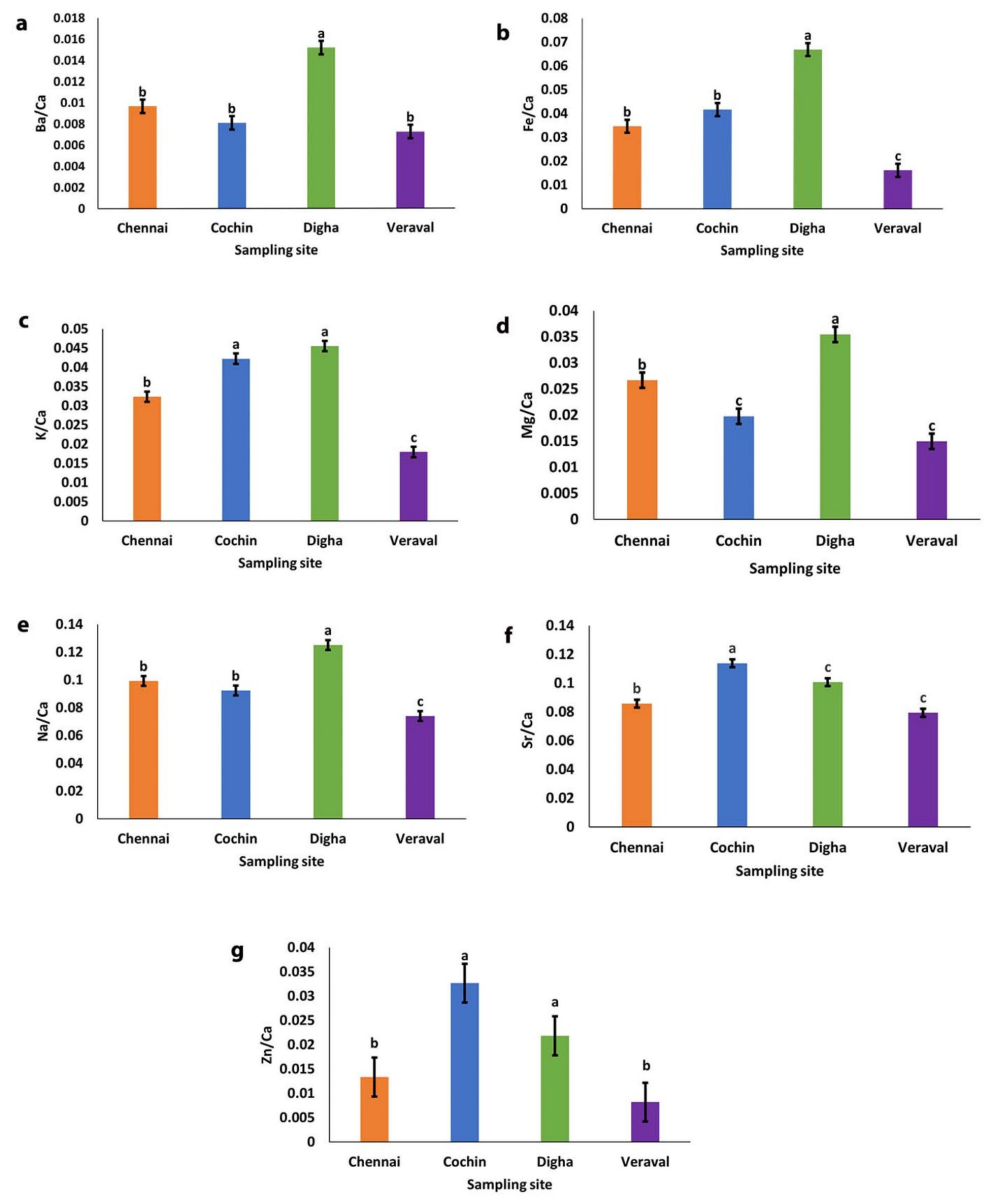
Location wise comparison of element/Ca ratios from the whole otolith of *D. russelli* from Cochin, Chennai, Digha and Veraval. (**a**) Ba/Ca ratios. (**b**) Fe/Ca ratios. (**c**) K/Ca ratios (**d**) Mg/Ca ratios. (**e**) Na/Ca ratios. (**f**) Sr/Ca ratios. (**g**) Zn/Ca ratios. Different letters above the boxes indicate significant differences between locations (Tukey’s, *p* < 0.0001).

We conducted the statistical analysis by grouping the data by coast (East and West) and by location (Chennai, Cochin, Digha and Veraval) to test the differences in elemental concentration at a finer geographical scale. All element/Ca ratios (Ba/Ca, Fe/Ca, K/Ca, Mg/Ca, Na/Ca, Sr/Ca, Zn/Ca) for the otolith differed significantly between the four sampling sites (one way ANOVA, *P* < 0.05) (Fig. 1; Table 1). It has been demonstrated that region explained the most variation in K/Ca and Fe/Ca (Table 1). On comparison of element/Ca ratios across location revealed that otoliths from Digha had significantly higher ratios for Ba/Ca, Fe/Ca, K/Ca, Mg/Ca and Na/Ca (Tukey’s, *p* < 0.05) (Fig. 1a, b, c, d and e) whereas otoliths from Cochin revealed significantly higher Sr/Ca and Zn/ Ca ratios (Tukey’s, *p* < 0.05) (Fig. 1f and g). All element/Ca ratios were significantly lower in the Veraval otolith (Tukey’s, *p* < 0.05) (Fig. 1). Further comparative analysis showed that, Ba/Ca, Mg/Ca and Na/Ca ratios were significantly higher in Chennai otoliths than in Cochin (Tukey’s, *p* < 0.05) (Fig. 1a, d, e) and Fe/Ca ratios were significantly higher in Cochin otoliths than in Chennai (Tukey’s, *p* < 0.05) (Fig. 1b). Furthermore, the Digha otolith demonstrated significantly higher Sr/Ca and Zn/Ca ratios (Tukey’s, *p* < 0.05) compared to the Chennai otolith (Fig. 1f and g).

**Table 1:** Location wise comparison of element/Ca ratios by one way ANOVA from the whole otolith of *D. Russelli* from Chennai, Cochin, Digha and Veraval.

Element	Source	df	MS	F value	*P* value	Estimates (%)
Ba/Ca	Region	3	0.00031710	31.72	< 0.0001	49.80
	Residual	96	0.00001000			50.20
	Total	99				
Fe/Ca	Region	3	0.01099730	58.13	< 0.0001	64.50
	Residual	96	0.00018919			35.50
	Total	99				
K/Ca	Region	3	0.00383140	83.80	< 0.0001	72.37
	Residual	96	0.00004572			27.63
	Total	99				
Mg/Na	Region	3	0.00197946	36.60	< 0.0001	53.36
	Residual	96	0.00005408			46.64
	Total	99				
Na/Ca	Region	3	0.01122134	36.73	< 0.0001	53.50
	Residual	96	0.00030548			46.60
	Total	99				
Sr/Ca	Region	3	0.00595638	31.80	< 0.0001	49.84
	Residual	96	0.00018729			50.52
	Total	99				
Zn/Ca	Region	3	0.00286299	7.20	0.0002	18.38
	Residual	96	0.00039739			81.62
	Total	99				

When we analysed the data by coast (east and west), it has been shown that, all element/Ca ratios were found to be significantly different between the coast (one way ANOVA, *p* < 0.05) with the exception of Sr/Ca (*p* = 0.380) and Zn/Ca (*p* = 0.515) (Supplementary Fig. S1 f and g). Ba/Ca, Fe/Ca, K/Ca, Mg/Ca and Na/Ca ratios were significantly higher on the east coast (Tukey’s, *p* < 0.05) (Supplementary Fig. S1 a, b, c, d and e). Region explained the most variation in Mg/Ca and Na/ Ca (Supplementary Table S1).

### Multi elemental analysis

Multivariate analysis (MANOVA) based on otolith chemistry demonstrated significant differences between all sampling sites (Wilk’s Lambda, *p* < 0.05; Pillai’s Trace, *p* < 0.05; Hotelling-Lawley Trace, *p* < 0.05; Roy’s Greatest Root, *p* < 0.05) (Table 2). It also revealed significant differences between the east and west coast of India (Wilk’s Lambda, *p* < 0.05; Pillai’s Trace, *p* < 0.05; Hotelling-Lawley Trace, *p* < 0.05; Roy’s Greatest Root, *p* < 0.05) (Supplementary Table S2).

**Table 2:** MANOVA test criteria and F approximations for overall location effect.

Statistic	Value	F Value	*P* value
Wilks’ Lambda	0.02908255	22.50	< 0.0001
Pillai’s Trace	1.76873808	14.37	< 0.0001
Hotelling-Lawley Trace	9.08194043	29.24	< 0.0001
Roy’s Greatest Root	4.84647871	48.46	< 0.0001

**Fig. 2:**
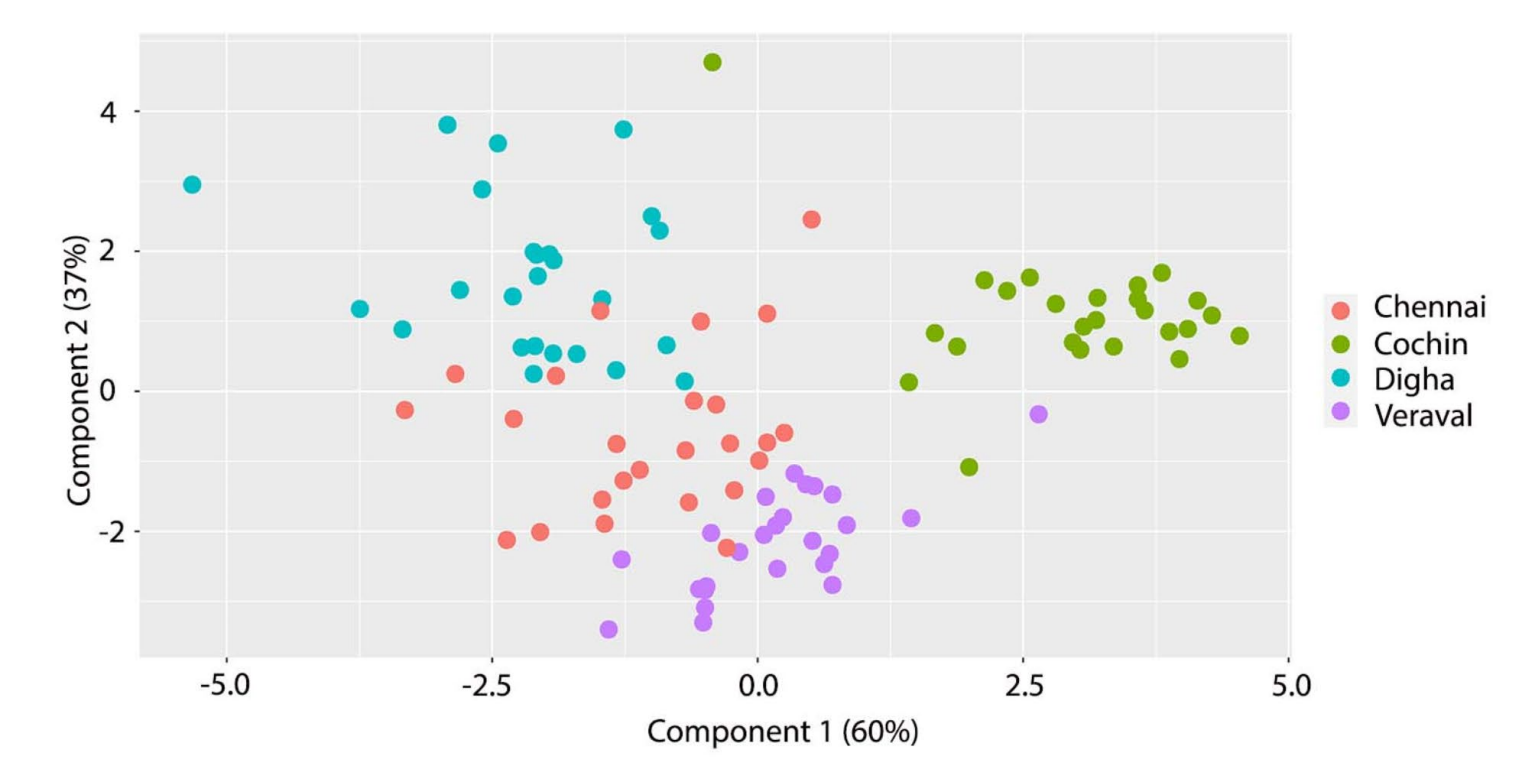
Plot of individual fish labelled by site, using variates derived from linear discriminant analysis of elemental ratios. Colored circles represent different locations (refer to legend).

The F approximations for all of the multivariate statistics in the Linear Discriminant Analysis (LDA) were significant with p values < 0.05, indicating that it is possible to distinguish between 4 sites based upon *D. russelli* otolith elemental composition. The first two axes of the LDA explained 97% of the variability, with the first axis being the more representative (60%) (Fig. 2; Table 3). Based on the coefficient of linear discriminants, the most relevant variable for classification were Na/Ca (-2.46) and Sr/Ca (1.70) for the first function and Na (-0.49) and K/Ca (1.39) for the second function (Table 3).

**Table 3:** Standardised coefficients and percent dispersion explained for the first two discriminant components (LD1 and LD2) in the analysis of otolith chemical signatures of *D. Russelli*.

	LD1	LD2
Ba/Ca	− 0.50864830	− 0.02453792
Fe/Ca	− 0.01063478	0.68050986
K/Ca	0.55108912	1.38804510
Mg/Ca	− 0.04406014	− 0.36318700
Na/Ca	− 2.45794559	− 0.48450698
Sr/Ca	1.69282975	0.61540761
Zn/Ca	0.06171334	0.08510018
Dispersion (%)	60	37

## Discussion

The chemical signatures of the otolith were used to distinguish *D. russelli* stocks for the first time in the present study. The analysis revealed significant spatial differences in otolith chemical composition among the four sampled populations, suggesting population heterogeneity.

*D. russelli* is a pelagic species known to undertake long distance migrations^53^, a behaviour that may facilitate genetic mixing among populations. Migratory fishes are generally considered to exhibit reduced population structure in marine environments as its present fewer barriers to dispersal allowing for extensive movement across marine habitats^54^. Several studies support this assumption, reporting low level of population structure in migratory species based on chemical^55^ and genetic analysis^16^. Nonetheless, there is conflicting data, with multiple studies utilizing genetic analyses^16–19^ and otolith chemical^29,56–59^ analyses to determine stock structure in migratory species. A number of additional studies revealed conflicting^27^ and supporting^55^ findings about the stock structure in marine species by using both otolith and genetic analyses concurrently.

The spatial variation observed in this study aligns with previous evidence of distinct stocks along the east and west coasts of India, as indicated by morphometric analysis^60^ and contrast with the panmictic nature of its population previously reported from genetic analysis across the Indian Ocean^16^. While otolith chemical (this study) and morphometric^60^ analyses suggest the presence of distinct stocks, the absence of genetic structure may be attributed to high levels of mixing and gene flow among populations. Such mixing could result from extensive breeding between populations, which may override any genetic differentiation that might otherwise emerge from spatial segregation. In addition, the species is reported to have an extended spawning season that overlaps along different coasts of India^7,9,10,15,61–63^. As a continuous breeder, the species takes advantage of favourable environmental conditions for spawning throughout its life, providing multiple opportunities for reproduction. If population mixing persists during the breeding season, genetic connectivity among populations may be maintained, explaining the apparent panmixia observed in genetic analysis.

Given the high migratory and dispersal capacity of Indian scad^53^, population homogeneity would be expected, as suggested by previous genetic analyses^16^. However, region-specific chemical signatures were observed in the whole otolith composition, with significant differences detected among the four sampling locations. Furthermore, five of the analysed elements exhibited significant differences between the east and west coasts. It has been reported that water chemistry is expected to fluctuate over time and space as chemical concentrations are influenced by various factors such as mixing of water masses, ion exchange between sediments and water, adsorption, complexation and precipitation^64^. Similarly, the chemical signatures in otoliths of individuals or groups of fish living in different environments can vary significantly. This variation depends on multiple factors, such as the duration of residence in a particular environment prior to capture (allowing for the integration of a detectable chemical tag), the physico-chemical properties of the habitat, and the specific elements selected for analysis^42^. Notably, such differences in chemical tags of fish groups across spatial and temporal scales can be used to infer connectivity between populations, even in the absence of direct information on the water chemistry of their habitats^42^.

The population heterogeneity observed in the present study for Indian scad can be attributed to the climatic phenomena and processes associated with different regions of the Indian Ocean. The tropical Indian Ocean plays a vital role in contributing the major part of the largest warm pool on Earth and its interactions with the atmosphere have a significant impact on both regional and global climate patterns^65^ This ocean system exhibits climatic variability on multiple temporal scales, ranging from seasonal cycles to interseasonal, interannual, and even longer-term fluctuations^65,66^: The strongest monsoon system on Earth is driven by the Asian continent, and the associated monsoon winds induce large seasonal shifts in ocean currents, including the Somali Current, the Southwest Monsoon Current (south of India), and the Northeast Monsoon Current (south of Sri Lanka). Unlike the Pacific and Atlantic Oceans, the Indian Ocean lacks equatorial upwelling. However, upwelling does occur in the Northern Hemisphere (off northwest Africa, the Arabian Peninsula, and east and west of the tip of India) and in the Southern Hemisphere (along the northern edge of southeastern waters). The shallow Equatorial Cross-Cell (CEC), which is unique to the Indian Ocean, facilitates equatorial heat transport and does not exist in other tropical oceans. Additionally, the Indian Ocean features an exchange route with the Pacific Ocean via the Indonesian Throughflow (ITF), which plays a crucial role in oceanographic connectivity and water mass exchange.

When examining specific regions of the Indian Ocean, notable differences in oceanic currents and water movement are observed. The southern Indian Ocean is characterized by the westward-flowing South Equatorial Current (SEC), which is primarily fed by the ITF. This current later bifurcates into northward and southward branches, serving as a major conduit for tropical-subtropical exchange and supplying water to the East African Coastal Current (EACC)^67^. The eastern Indian Ocean is distinguished by an intensified eastward-flowing zonal current that expands latitudinally as it moves eastward. This region also features the East Gyral Current (EGC), a geostrophic eastward flow linked to the ITF and fed from the north^68^. The northern Indian Ocean is dominated by the northward-flowing Somali Current, which is supplied by the SEC and EACC during the summer monsoon^69^. As it flows, the Somali Current forms various gyres and eddies, including the Southern Gyre near the equator, the Great Whirl to the north, and the Socotra Eddy in the northeast. It also generates a cold upwelling wedge after crossing the equator. During the winter monsoon, the Somali Current reverses its direction, flowing southward to meet the EACC, which, in turn, feeds the eastward-flowing South Equatorial Counter Current^70^. These inter oceanic flows and upwelling wedges associated with these currents can have an impact on surface wind stress and heat fluxes^71^. Such factors can alter water chemistry at sampling sites, thereby contributing to region-specific geochemical signatures. Previous studies have reported that these physical processes affect the elemental composition of fish otoliths, providing insights into regional geochemical differences or similarities among fish populations^42,43,72,73^.

Our findings also suggested that environmental processes can influence water chemistry, which, in turn, affects the elemental composition of calcified structures like otoliths. In our study, the elements such as barium (Ba), iron (Fe), magnesium (Mg), strontium (Sr), and zinc (Zn), which are reportedly under strict environmental regulation^74–76^ significantly contributed to stock discrimination among the four sampling sites. Notably, magnesium (Mg) played a key role in distinguishing populations between the two coasts, contributing a higher percentage to the classification. Previous studies have shown that the concentration of strontium (Sr) in fish otoliths is significantly influenced by its ambient concentration in the water^58^. In addition, it is known that there is a positive relationship between Sr/Ca and water salinity^40,41,77^. In the present study, the higher Sr/Ca ratios observed in the Digha and Cochin otoliths could be attributed to the influence of the southwest monsoon currents, which transport the saltier waters of the Arabian Sea into the Bay of Bengal^40^. Barium (Ba) is widely regarded as a reliable indicator of the upwelling process and flow of ocean currents and associated changes in water chemistry. As stated above, in the northwest Indian Ocean, upwelling events occurring during the southwest monsoon generate an upward movement of nutrient rich waters from below the thermocline to shallower depths, which possibly implies the observed higher Ba/Ca concentrations in the whole otolith in the region. Additional oceanographic features, such as intensified zonal eastward flow^70^, the eastward geostrophic flow, EGC^68^ and the Socotra Eddy Cell of the Somali current^70^ may also contribute to the observed patterns in Ba/Ca ratios. Moreover, prior research has demonstrated a positive correlation between Ba/Ca ratios and salinity^78^, which aligns with our findings. A notable relationship between Sr and Ba has been reported, suggesting that elevated Sr concentrations may facilitate the uptake of Ba into fish otoliths^79^. Similarly, a positive correlation between magnesium (Mg) and salinity has been documented^80^, further supporting the results of this study.

There is no proof that the physiochemical characteristics of the surroundings affect the integration of Fe. Previous studies found no correlation between the temperature or salinity of the surrounding water and Fe uptake in otoliths^77^. Zn uptake is known to have an inverse relationship with salinity^77^. Although the Arabian Sea is generally characterized by high salinity, the westward flow of the northeast monsoon current transports the fresher waters of the Bay of Bengal into the Arabian Sea, thereby reducing salinity. This process likely explains the higher Zn/Ca ratios observed in the Cochin otoliths. In addition, Wyrtki Jets (WJs), which occur only in the Indian Ocean, transport warm upper layer water eastward, causing sea level and mixed layer thickness to rise in the east and decrease in the west^66^. This remarkable Indian Ocean phenomenon could be responsible for the exceptionally low concentration of all element/Ca ratios in the Veraval otolith and also for the exceptionally higher concentration of elements on the east coast, since almost all of the elements we discussed have a positive correlation with temperature^41,77,80^. The remaining elements K and Na are reported to be essential for cellular processes and have an additional physiological response to their regulation in addition to environment^74^.

The findings of this study have significant implications for the management, conservation, and sustainable exploitation of *D. russelli* stocks in the Indian Ocean. The ability to identify distinct fish stocks using otolith chemical analysis provides a powerful alternative to traditional genetic analysis, offering critical insights into population structure and connectivity. Unlike genetic analysis, which reflects long-term evolutionary connectivity, otolith chemistry captures short-term ecological connectivity, making it a valuable tool for monitoring the movement and residency of fish in different marine habitats. This information is vital for the development of region-specific fisheries management plans, as it highlights the potential presence of discrete fish stocks along the east and west coasts of India.

The observed spatial variation in elemental composition of otoliths underscores the influence of environmental factors, such as ocean currents, upwelling, and monsoonal shifts, on population structure. Identifying these region-specific chemical signatures allows for the demarcation of biologically distinct stocks, which can be used to establish more effective fishery management units. This is especially critical for highly migratory species like *D. russelli*, where panmictic populations are often assumed but not always supported by ecological markers like otolith chemistry. Mismanaging such stocks under the assumption of homogeneity could lead to overfishing or localized depletion of specific populations.

Additionally, the integration of otolith chemistry with other complementary methods, such as morphometric and genetic analyses, provides a more holistic approach to stock discrimination. The combined use of multiple markers increases the accuracy and reliability of stock assessments, reducing the risk of misclassification and enabling more informed management decisions. This integrative approach is crucial for addressing the complexity of marine stock structure, especially in the context of changing environmental conditions driven by climate variability.

Moreover, the study’s demonstration of the environmental control of elemental uptake into otoliths highlights the potential of using otolith chemistry as a biomonitoring tool for tracking climate-driven changes in ocean conditions. Given the unique climatic and oceanographic features of the Indian Ocean, such as Wyrtki Jets, the Somali Current, and the influence of monsoonal currents, otolith chemical signatures could provide early warning indicators of environmental shifts. This is particularly relevant for assessing the impacts of climate change on marine ecosystems and understanding how changes in sea temperature, salinity, and water mass mixing affect marine biodiversity and fish population dynamics.

Overall, the study emphasizes the need for region-specific fishery management strategies that recognize the presence of multiple, spatially distinct stocks within *D. russelli* populations. The use of otolith chemistry as a tool for stock assessment and connectivity analysis can improve the precision of stock management, contribute to sustainable fishing practices, and support the long-term conservation of this ecologically and economically important species.

## Conclusion

This was the first study in this species which assessed the otolith chemical composition for stock structure analysis. The present study reported significant differences in elemental composition among the four sampling sites. Five of the seven element/Ca ratios contributed to the differentiation among coast with the east coast providing the largest contribution of the overall elemental composition. These variations may be driven by regional oceanographic factors, such as near-surface circulation and upwelling in different areas of the Indian Ocean, supporting the notion of population heterogeneity within *D. russelli*. The results of the present study provide new information on the spatial distribution of *D. russelli* that contradicts the previous suggestion based on genetic analysis that the species is panmictic. The observed lack of genetic structure for *D. russelli* by the present authors^16^ is not necessarily inconsistent with the findings from otolith chemical analysis. Instead, it emphasizes the importance of integrating both ecological connectivity (as revealed by otolith chemistry) and evolutionary connectivity (as indicated by molecular genetic markers) to better understand systems with extremely low levels of population structure. consequently, this study also highlights the discriminatory capacity of various methods and the need to use multiple approaches to unravel the spatial ecology of highly migratory fish species.

This basic information on ecology and population structure is crucial as a basis for formulating effective management and conservation strategies for the species. Future studies using data collected along a complete ontogenetic transect of the otolith is necessary to examine whether individuals migrate to the same location for feeding and breeding throughout their lives to distinguish between the mechanism of genetic homogenization and the ecological differentiation. In addition, further sampling during the species’ breeding season may provide sufficient information about whether the population structure is present. If individuals return to the same location to breed every year, there should be a strong population structure at the genetic level, which unfortunately is not the case for *D. russelli*^16^. In addition, sampling before and after the breeding season can also help examine the structure of the population at a finer scale.

## Materials and methods

### Study area and sample collection

**Fig. 3:**
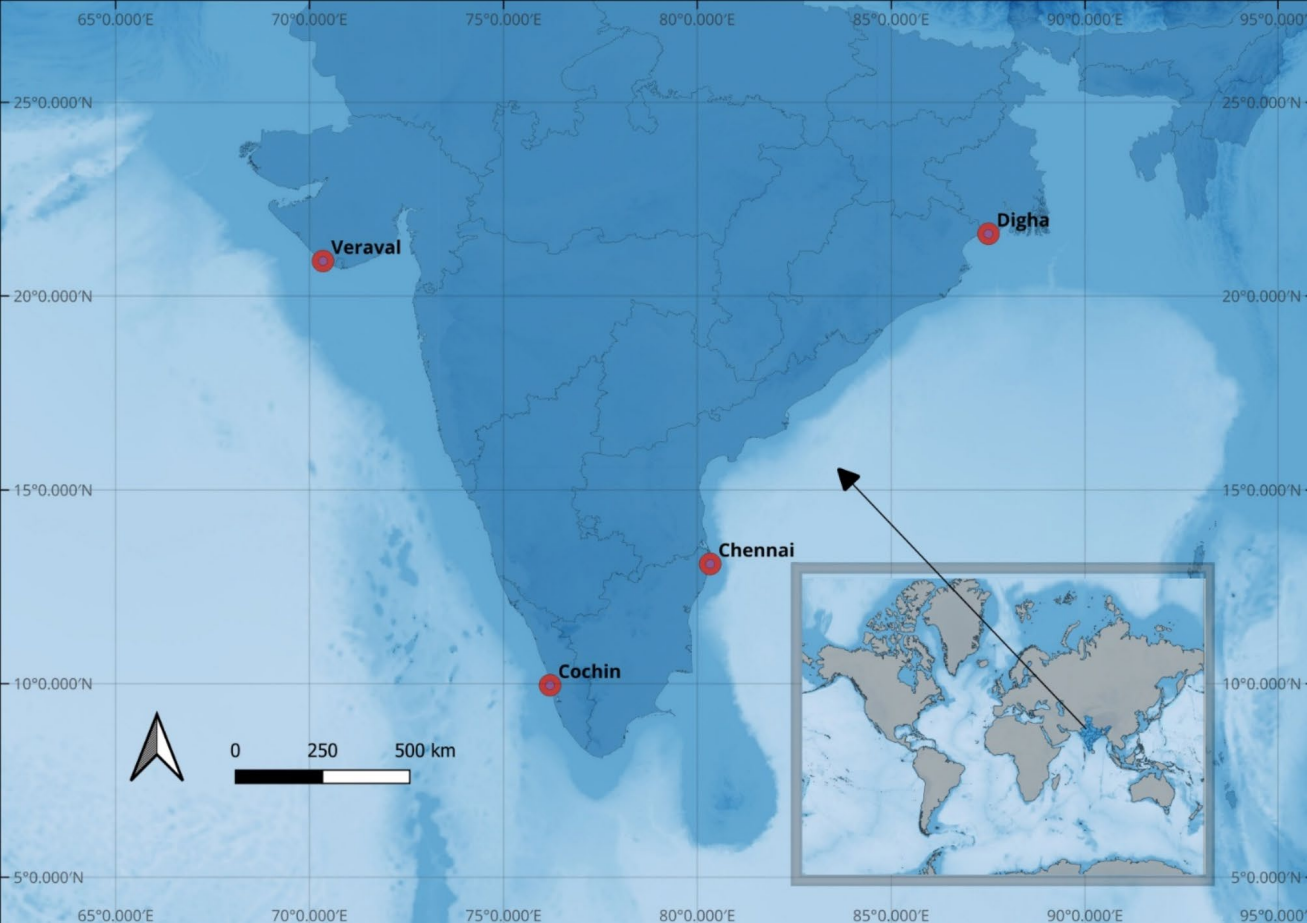
Map indicating sampling locations of *D. russelli* along Indian Ocean.

In 2023, *D. russelli* specimens were collected from four locations along the Indian coast between January and March. The sampling sites included two locations on the east coast (Digha and Chennai) and two on the west coast (Veraval and Cochin) of India (Fig. 3). The locations were chosen to align with the previous genetic analysis conducted^16^, facilitating a comparison to corroborate the genetic findings. Twenty-five specimens were collected from each site using trawl and purse seines operating near the coast. Whole fish were preserved frozen in the field and returned to the laboratory for processing. The length (TL) (cm) of fish was recorded before preparing the otolith for subsequent analysis (Supplementary Table S3). The fish sample used in this study was treated in accordance with the recommendations made by De Tolla et al.^81^ for the handling and use of fish in research. The protocols were approved by the ethical committee of the ICAR- Central Marine Research Institute, Kochi. These methods are also reported following ARRIVE guidelines (http://arriveguidelines.org).

### Otolith preparation

**Fig. 4:**
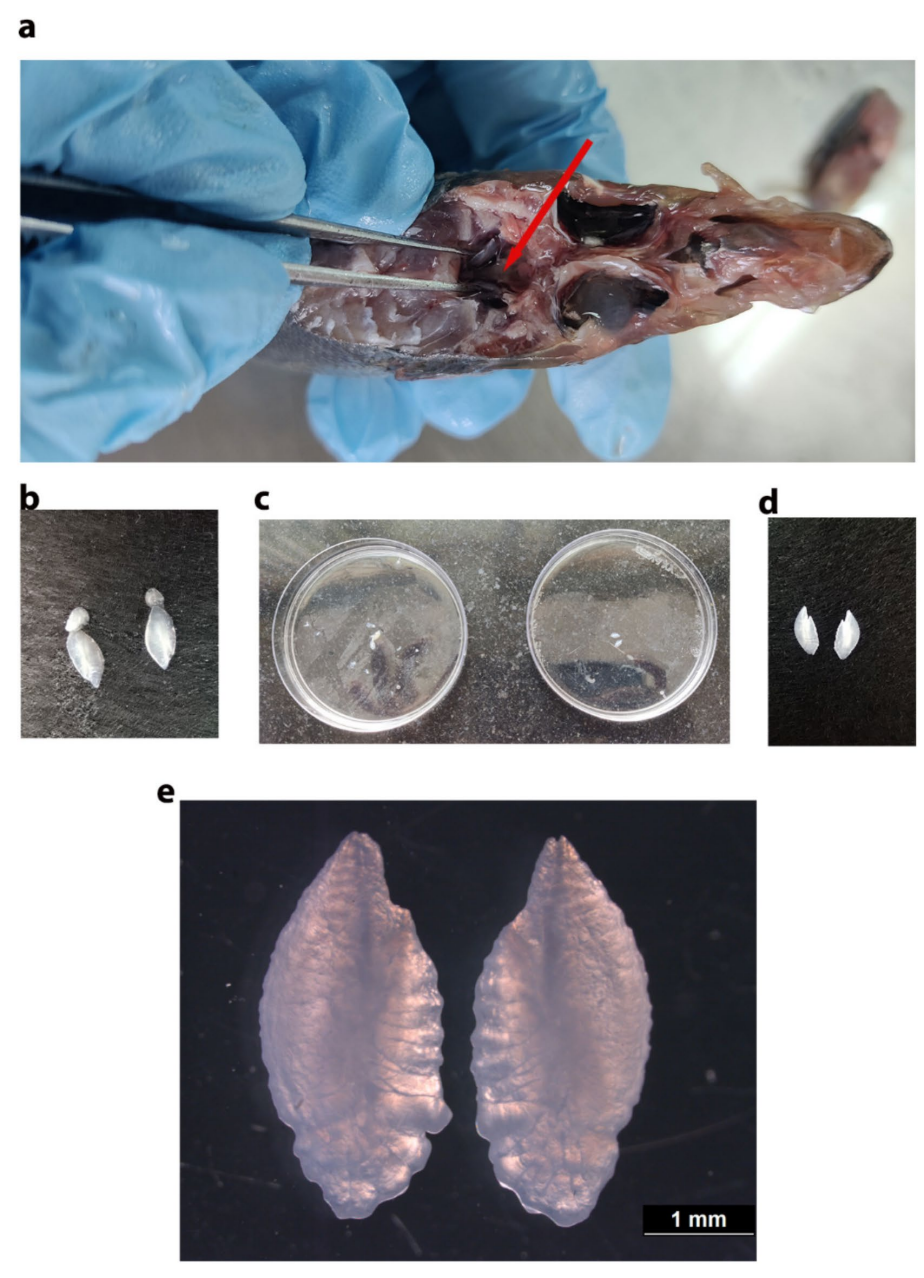
Removal and preparation of otolith for elemental analysis (**a**) Removal of otolith by open the hatch method; red arrow indicates the location of the otolith. (**b**) All the three pairs of otolith and adherent tissue. (**c**) Washing and (**d**) cleaning of sagittal otolith (**e**) Stereo zoom image of a pair of sagittal otoliths from *D. russelli*.

A pair of sagittal otoliths were removed by open the hatch method^82^ (Fig. 4) from the 25 specimens from each study site. All adhering tissues were removed using an acid washed nylon brush and forceps. The otoliths are then decontaminated by rinsing with Milli-Q water and then completely air dried. Following decontamination, the otolith was stored until processing in vials which are previously washed with dilute nitric acid and ultra distilled water and oven dried. Before storage the otolith weight (g) was recorded for each individual (Supplementary Table S3).

### Otolith chemical analysis

A pair of sagittal otoliths from each individual are first digested with 4 ml of 1–2% trace metal grade nitric acid (TraceMetal™ Grade, Fisher Scientific) and 1 ml of 30–32% H_2_O_2_ (Optima™, Fisher Scientific) using Milestone Start D microwave extraction system (Milistone s.r.l., Sorisole, Italy), equipped with easy CONTROL software and HPR 1000/10S high pressure segmented rotor at a pressure of 400 psi and power of 1200 W. The conditions for sample digestion were set as follows: ramp upto 180 °C for 20 min; hold at 180 °C for 20 min and cooled down for 10 min at room temperature. Prior to analysis, the digested samples were filtered using a 0.45 micron syringe filter and diluted to known volume with ultra-pure water. Microwave condition and digestion procedures were followed according to Milestone Cookbook Digestion Rev. 03_04. Temperature and pressure sensors were used to monitor digestion conditions. The elemental analysis of *D. russelli* otolith samples were carried out using Inductivity Coupled Plasma–Optical Emission Spectrometer (ICP-OES) (iCAP 6300 Duo, Thermo fisher Scientific, Cambridge, England) with dual configuration (axial and radial) and iTEVA operational software (version 2.8.0.97). The instrumental conditions used were: optics temperature 38 °C, camera temperature of -44 °C; Nebulizer (MiraMist Cyclonic Chamber) main argon flow rate of 15 L per min, auxiliary argon flow rate of 0.5 L per min, gas flow of 0.5 L per min and maximum integration time of 30 s. ICP multi-element standard solution (CentiPUR, Merck-Supelco^®^, Mumbai, India) was used for the preparation of calibration solutions. Five calibration levels of multi-element standard ranging from 0.2 to 2 mg/kg were prepared by dilution with 1% HNO3. The samples were analysed for Barium (Ba), Iron (Fe), Magnesium (Mg), Potassium (K), Sodium (Na), strontium (Sr), Zinc (Zn) and reported as ratios to calcium (Ca). The mean concentration (ppm) of each element from the four-sampling location detected via ICP-OES is shown in Table 4. Test value specified for the acid are ≤ 1 µg/Kg in case of Ca, Fe, K, Mg and Na, whereas ≤ 0.1 µg/Kg for Ba and Sr, ≤ 0.5 µg/Kg for Zn. Reagent blanks were analysed along with digested samples to confirm any presence of contamination. The method for analysis of metals in fish using ICP-OES is validated as per requirement of AOAC Guidelines for Standard Method Performance Requirements. LOQ of Ca, Fe, K, Mg, Na, Zn, Ba and Sr are 20, 5, 30, 6, 40, 1.5, 0.25 and 0.5 mg/Kg respectively.

**Table 4:** Summary table of the ICP-OES data (concentration in ppm) for all four study sites.

Sl. No.	Element	Chennai	Cochin	Digha	Veraval
		Samples (*n* = 25)	Samples (*n* = 25)	Samples (*n* = 25)	Samples (*n* = 25)
		Average ± SD	Average ± SD	Average ± SD	Average ± SD
1	Ba	25.973 ± 7.70	14.298 ± 14.01	57.680 ± 35.38	17.999 ± 18.23
2	Ca	2,79,681 ± 54,493	2,20,376 ± 202,507	2,37,155 ± 11,309	2,51,396 ± 86,674
3	Fe	389.684 ± 313.57	361.852 ± 192.34	1168.902 ± 715.59	64.1597 ± 33.70
4	K	296.376 ± 147.53	347.148 ± 143.32	498.626 ± 11.26	84.9862 ± 45.00
5	Mg	204.852 ± 87.85	81.002 ± 48.28	37.753 ± 302.09	60.652 ± 40.16
6	Na	2988.633 ± 1457.72	1657.52 ± 582.61	3730.206 ± 406.54	1574.301 ± 1010.53
7	Sr	2162.252 ± 855.86	2435.88 ± 199.89	2407.852 ± 155.54	1744.03 ± 990.05
8	Zn	87.0468 ± 209.29	132.423 ± 63.25	120.183 ± 67.14	36.645 ± 117.20

### Statistical analyses

All statistical analyses were performed using PROC GLM of SAS software (Ver. 9.3). Spearman’s rank correlation coefficient was worked out to find the degree of linear relationship of the element/Ca ratios with the total length (TL) of fish and otolith weight (W) according to the fulfilment of the normality and homogeneity assumptions. No significant correlation was found between TL or W and elemental ratios (Supplementary Table S4). The data collected on element/Ca ratios in otoliths from different locations/regions were subjected to one-way Analysis of Variance (ANOVA) to find the significant difference among the locations/regions. Further, a multiple comparison among the locations/regions were made using Tukey’s HSD test. The significant difference between locations/regions were depicted by different alphabets. The data were transformed by using square root transformation to make it amenable to ANOVA. The significance was assessed at *p* < 0.05. A Multivariate ANOVA (MANOVA) was performed to assess the significance among the study sites/regions while considering all the element/Ca ratios simultaneously based on four statistical tests viz., Wilk’s Lambda, Pillai’s Trace, Hotelling-Lawley Trace and Roy’s Greatest Root). Linear Discriminant Analysis (LDA) was performed using all 100 observations to determine if study sites could be discriminated based upon the elemental composition of otoliths using the R software^83^ after standardizing the variables (element/Ca ratios). An LDA plot was also drawn using the first two linear discriminant function scores to depict the classification of samples.

## Electronic supplementary material

Below is the link to the electronic supplementary material.

Supplementary Material 1

## Data Availability

The data that support the findings of this study are available on request from the corresponding author.
