# Supplementary material for: Otolith chemistry suggests population heterogeneity within a genetically homogeneous Indian scad population along Indian coast
Source: Sci Rep. 2025 Jan 8;15:1335. doi: 10.1038/s41598-025-85719-3 (PMC11711401; doi:10.1038/s41598-025-85719-3)
Supplement: Supplementary file 1 — Supplementary Material 1 [file 41598_2025_85719_MOESM1_ESM.pdf]

Otolith chemistry suggests population heterogeneity within a genetically homogeneous Indian scad population along Indian coast

Anjaly Jose[1,2], Sandhya Sukumaran[1*], Neenu Raj[1], Nisha K[1], Eldho Varghese[1], Laly S.J[3], Satyen Kumar Panda[3], Subal Kumar Roul[1], Abdul Azeez P[1], Shoba Joe Kizhakudan[1] and A. Gopalakrishnan[1]

[1]Marine Biotechnology Fish Nutrition and Health Division, ICAR-Central Marine Fisheries Research Institute, Ernakulam North P O, Kochi, Kerala, India-682018
[2]Mangalore University, Mangalagangotri, Mangalore, Karnataka, India-574199
[3]ICAR-Central Institute of Fisheries Technology, Kochi, Kerala, India-682029
*Corresponding author
Email address: sandhyasukumarancmfri@gmail.com

Supplementary Table S1. Coast wise comparison of element/Ca ratios by one way ANOVA from the whole otolith of *D. russelli* from east and west coast of Indian Ocean

**Element**	**Source**	**df**	**MS**	**F value**	**P value**	**Estimates (%)**
	Region	1	0.00056426	41.06	<0.0001	29.52
Ba/Ca	Residual	98	0.00001374			70.48
	Total	99				
	Region	1	0.01192254	29.78	<0.0001	23.30
Fe/Ca	Residual	98	0.00040033			76.70
	Total	99				
	Region	1	0.00195077	13.72	0.0004	12.30
K/Ca	Residual	98	0.00014217			87.70
	Total	99				
	Region	1	0.00468187	71.16	<0.0001	42.07
Mg/Na	Residual	98	0.00006580			57.93
	Total	99				
	Region	1	0.02104492	49.17	<.0001	33.40
Na/Ca	Residual	98	0.00042801			66.60
	Total	99				
	Region	1	0.00028224	0.78	0.3800	0.80
Sr/Ca	Residual	98	0.00036292			99.20
	Total	99				
	Region	1	0.00020267	0.43	0.5151	0.44
Zn/Ca	Residual	98	0.00047485			99.56
	Total	99				

Supplementary Table S2. MANOVA test criteria and F approximations for overall coast effect

Statistic	Value	F Value	P value
**Wilks' Lambda**	0.30910326	22.35	<.0001
**Pillai's Trace**	0.69089674	22.35	<.0001
**Hotelling-Lawley Trace**	2.23516487	22.35	<.0001
**Roy's Greatest Root**	2.23516487	22.35	<.0001

Supplementary Table S3. Descriptive statistics of *D. russelli* samples used for otolith chemical analysis. N=Number of samples, TL=total length (cm), W=otolith weight (g), SD=standard deviation.

Sampling site	N	TL ± SD	W±SD
Chennai	25	16.704 ± 1.23	0.013113 ± 0.002
Cochin	25	18.136 ± 0.97	0.015068 ± 0.002
Digha	25	16.296 ± 0.79	0.010896 ± 0.001
Veraval	25	16.604 ± 1.25	0.014108 ± 0.002

Supplementary Table S4. Spearman rank correlation coefficients for the relationship between TL and otolith weight on element/Ca ratios. P values are provided in brackets. TL=total length, W=otolith weight.

	Ba/Ca	Fe/Ca	K/Ca	Mg/Ca	Na/Ca	Sr/Ca	Zn/Ca
TL	-0.2305 (0.0210)	0.0089 (0.9298)	0.0166 (0.8695)	-0.2069 (0.0388)	-0.1983 (0.0479)	0.3849 (<.0001)	0.3058 (0.0020)
W	-0.4578 (<.0001)	-0.3950 (<.0001)	-0.3081 (0.0018)	-0.4776 (<.0001)	-0.4828 (<.0001)	0.0877 (0.3855)	0.0095 (0.9248)

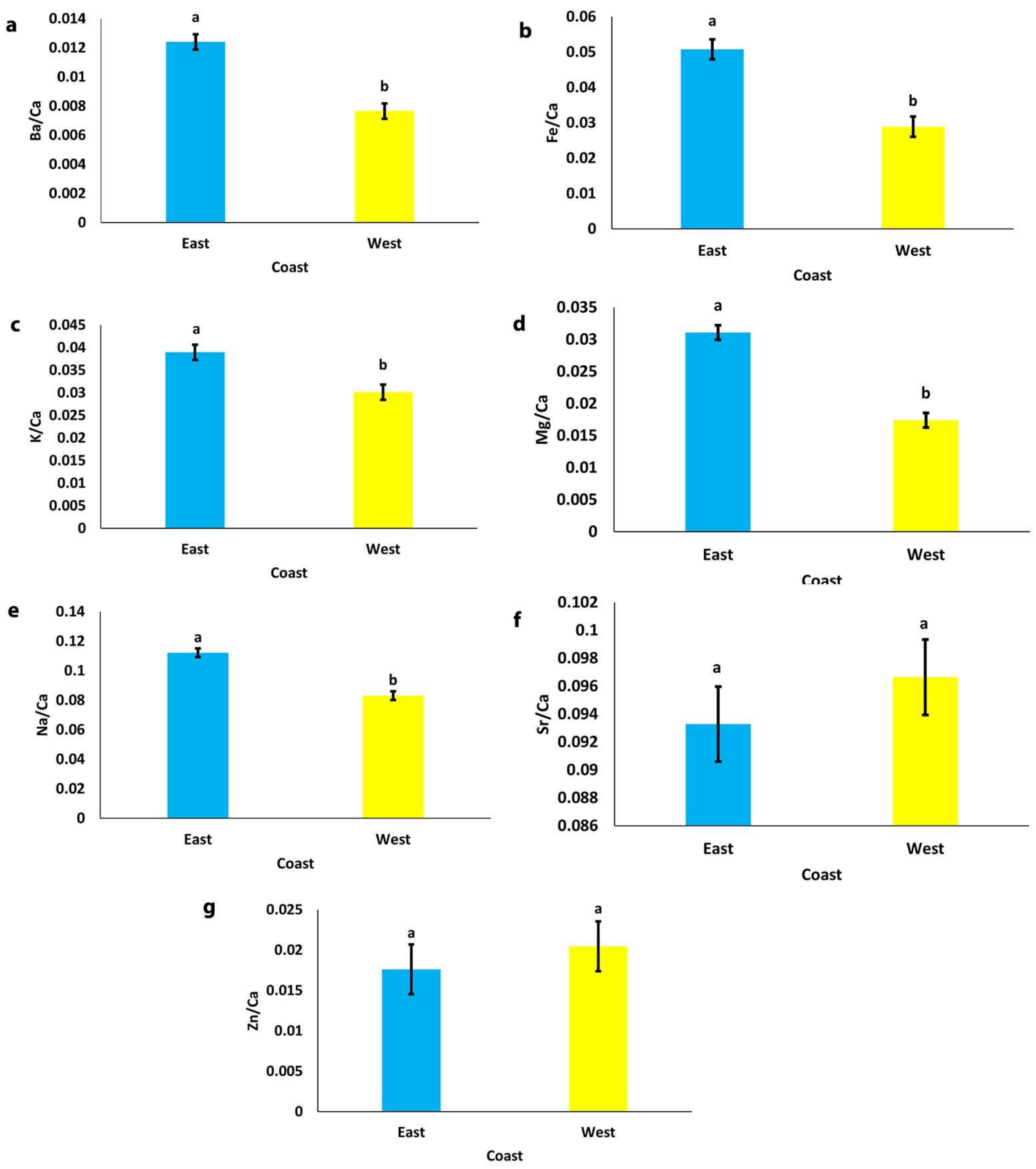

Supplementary Figure S1. Coast wise comparison of element/Ca ratios from the whole otolith of *D. russelli* from east and west coast of Indian Ocean. (a) Ba/Ca ratios. (b) Fe/Ca ratios. (c) K/Ca ratios (d) Mg/Ca ratios. (e) Na/ Ca ratios. (f) Sr/Ca ratios. (g) Zn/Ca ratios. Different letters above the boxes indicate significant differences between coast (Tukey's, $p < 0.0001$).
